# Supplementary material for: Resolving the full spectrum of human genome variation using Linked-Reads
Source: Genome Res. 2019 Apr;29(4):635–45. doi: 10.1101/gr.234443.118 (PMC6442396; doi:10.1101/gr.234443.118)
Supplement: Supplemental Material [file supp_gr.234443.118_Supplemental_longranger-2.2.zip › longranger-2.2.tar.bz2!/lr2.2-public/lib/go/src/code.google.com/vitess/docs/user-guide/mysql-server-protocol.html]

Vitess / MySQL Server Protocol


Toggle navigation

- Overview
- Guides
- Reference
- Blog
- GitHub

- #### Overview

  - What is Vitess
  - Scaling MySQL with Vitess
  - Key Concepts
- #### Getting Started

  - Run Vitess on Kubernetes
    - Custom Docker Build
  - Run Vitess Locally
- #### User Guide

  - Introduction- Client Libraries- Backing Up Data- Reparenting
        - Schema Management
        - VSchema Guide
        - Schema Swap (Tutorial)
        - Sharding
          - Horizontal Sharding (Tutorial, automated)
          - Horizontal Sharding (Tutorial, manual)
          - Sharding in Kubernetes (Tutorial, automated)
          - Sharding in Kubernetes (Tutorial, manual)
        - Vitess Sequences
        - MySQL Server Protocol
        - Vitess and Replication
        - Update Stream
        - Row Based Replication
        - Topology Service
        - Transport Security Model
        - Launching
          - Scalability Philosophy
          - Production Planning
          - Server Configuration
          - 2PC Guide
          - Troubleshooting
        - Upgrading
- #### Reference Guides

  - Vitess API- vtctl Commands
- #### Other Resources

  - Presentations- Blog- Roadmap
- #### Contributing

  - Contributing to Vitess- GitHub Workflow- Code Reviews
- GitHub

# MySQL Server Protocol

- #### Overview

  - What is Vitess
  - Scaling MySQL with Vitess
  - Key Concepts
- #### Getting Started

  - Run Vitess on Kubernetes
    - Custom Docker Build
  - Run Vitess Locally
- #### User Guide

  - Introduction- Client Libraries- Backing Up Data- Reparenting
        - Schema Management
        - VSchema Guide
        - Schema Swap (Tutorial)
        - Sharding
          - Horizontal Sharding (Tutorial, automated)
          - Horizontal Sharding (Tutorial, manual)
          - Sharding in Kubernetes (Tutorial, automated)
          - Sharding in Kubernetes (Tutorial, manual)
        - Vitess Sequences
        - MySQL Server Protocol
        - Vitess and Replication
        - Update Stream
        - Row Based Replication
        - Topology Service
        - Transport Security Model
        - Launching
          - Scalability Philosophy
          - Production Planning
          - Server Configuration
          - 2PC Guide
          - Troubleshooting
        - Upgrading
- #### Reference Guides

  - Vitess API- vtctl Commands
- #### Other Resources

  - Presentations- Blog- Roadmap
- #### Contributing

  - Contributing to Vitess- GitHub Workflow- Code Reviews

# MySQL Binary Protocol

Vitess 2.1 is adding alpha support for the MySQL binary protocol (refered to as the
`protocol` in the rest of this document). This allows existing applications to
connect to Vitess directly without any change, or new driver / connector.

However, this also has limitations. The RPC protocol traditionnally exposed by
Vitess is reacher in features, and allows finer grain control of the query
behaviours.

This document explores the limitations of using this protocol.

## Protocol Limitations

The following features are not represented in the protocol.

### Bind Variables

In the traditional connector, queries are sent as plain text to the server. This
protocol does not support bind variable.

The Prepared Statement part of the API can support bind variables, but it
requires per-connection state, and using binary bind variables, which are much
harder to implement. It is also not recommended.

Most database drivers (JDBC, go, ...) support bind variables. These end up being
implemented as client-side bind variables, where the values are printed in the
SQL statement by the client, and re-interpreted by the server.

A Vitess Connector, on the other end, can send the Bind Variable map to the
server along with the query. The query plan is then cached by both vtgate and
vttablet, providing much better execution times.

Note we added the `normalize_queries` command line parameter to vtgate to
mitigate this problem. With this flag, vtgate will try to extract bind variables
from full queries. This makes the vttablet side optimized, but still costs extra
CPU on the vtgate side.

### Tablet Type

The regular Vitess API provides the ability to specify the tablet type to
target: `master`, `replica`, `rdonly`. The current MySQL connector we created
only uses the `master` type.

Note we could implement a different policy for this.

### Transaction Type

The regular Vitess API provides the ability to specify the transaction type: one
shard only, or 2PC. The current MYSQL connector uses the transaction type
provided to vtgate by the `transaction_mode`, which usually is the highest
transaction level allowed by vtgate, not the default one.

### Streaming Queries

The Vitess RPC protocol supports both non-streaming queries (for web-like
traffic), and streaming queries (for data analysis traffic). The current MySQL
connector only uses non-streaming queries.

This could be changed with a flag. Or we could try to be smart: anything within
a transaction could be non-streaming query, anything outside a transaction could
be streaming.

### Extra Query Features

There are even more Vitess specific features in the API, that are not
represented in the MySQL server API. Event Tokens for cache invalidation, Update
Stream, Messages, ... These seem somewhat impossible to implement using the
MySQL binary protocol.

### Security

The current RPC protocol uses the security provided by the RPC protocol, TLS for
gRPC. We then use the certificate name as the user name for our authorization
(table ACLs).

The MySQL server connector requires user names and passwords, that need to
maintained, rotated, ... We do not include TLS support yet, although it can be
easily added. We also need to add the username as authenticated user to use for
our authorization.

We have plans to provide an LDAP plug-in to authenticate the users (but no firm
development plan for it yet).

### Query Multiplexing

gRPC can multiplex multiple request / responses on the same TCP connection. The
MySQL server protocol is very synchronous, and can only have one request in
flight at any given time.

## Recommended Use Cases

With all these limitations, why did we even bother implementing this? Well,
there is something to be said for drop-in replacement and ease of use:

- When migrating an existing application to Vitess, it is useful to run Vitess
  on top of an existing database, and only change the server address in the
  client code to point at vtgate, and not change anything else. This allows for
  an easier transition, and then the client code can be update later to use a
  better connector.
- Some tools only support MySQL server protocol, and not connectors / not Vitess
  (yet!).
- Most of the mentioned limitations are going to affect production systems
  running at a somewhat high load. For smaller workloads, none of this really
  matters.
- A lot more programming languages have MySQL connectors than what we support
  already (we have connectors for Java, Python, PHP, Go). They can use the MySQL
  server protocol until a native connector for gRPC can be immplemented.

## Future Features

We are thinking about the following features to make this connector more useful:

- Add SSL support.
- Provided more authentication schemes than `mysql_native_password`. In
  particular, `sha256` seems easy.
- Use an LDAP client to validate users and passwords on the server side.
- Implement a lot more DBA features in the protocol. Statements like `SHOW
  TABLES` are not supported yet. They will make the MySQL binary protocol much
  more useful.
- Possibly add hints to the queries so they can use more advanced features. Like
  provide a `commit 2pc` syntax to enable 2PC for some commits.
- Provide direct SQL access for some features like Vitess Messages.

Contact: vitess@googlegroups.com  **·**  
Announcements  **·**  
© 2017 Vitess powered by Google Inc
